# Study on the prevalence of soil contamination with *Ancylostoma* spp. eggs in public parks and playgrounds in Hue City, Vietnam

**DOI:** 10.1590/S1984-29612026026

**Published:** 2026-07-20

**Authors:** Ho Thi Dung, Cai Nguyen Hoang Ngan, Nguyen Thi Hoa, Pham Hoang Son Hung

**Affiliations:** 1 Hue University of Agriculture and Forestry, Hue University, Hue city, Vietnam

**Keywords:** Cutaneous larva migrans, environmental contamination, epidemiology, parasite, Larva migrans cutânea, contaminação ambiental, epidemiologia, parasito

## Abstract

This study aimed to assess soil contamination with *Ancylostoma* spp. eggs in public parks and playgrounds in Hue City. A total of 240 soil samples were collected from 60 public parks and playgrounds located in two urban zones (central urban and peri-urban areas) during both the rainy and dry seasons. *Ancylostoma* spp. eggs were detected at 48 of 60 sampling sites (80%). The proportion of contaminated sites was significantly higher in central urban areas compared with peri-urban areas and during the rainy season compared with the dry season. In contrast, no significant differences were observed between sampling locations within individual parks, including areas near garbage bins and playground areas. Most positive sites showed low contamination intensity, with 1–4 eggs per 200 g of soil. Conventional PCR confirmed the presence of *Ancylostoma caninum* DNA in positive soil samples, indicating that dogs are the primary source of environmental contamination. These findings provide baseline data on soil-transmitted hookworm contamination in Hue City and highlight the need for improved dog faeces management, ecological sanitation, and public health education to reduce the risk of community exposure.

## Resumo

Este estudo teve como objetivo avaliar a contaminação do solo por ovos de *Ancylostoma* spp. em parques públicos e playgrounds da cidade de Hue. Foram coletadas 240 amostras de solo em 60 parques públicos e playgrounds localizados em duas zonas urbanas (área urbana central e áreas periurbanas), durante as estações chuvosa e seca. Ovos de *Ancylostoma* spp. foram detectados em 48 dos 60 locais amostrados (80%). A proporção de locais contaminados foi significativamente maior nas áreas urbanas centrais do que nas periurbanas, bem como durante a estação chuvosa em comparação com a estação seca. Em contrapartida, não foram observadas diferenças significativas entre os locais de amostragem dentro de cada parque, incluindo áreas próximas a lixeiras e áreas de playground. A maioria dos locais positivos apresentou baixa intensidade de contaminação, com 1–4 ovos por 200 g de solo. A PCR convencional confirmou a presença de DNA de *Ancylostoma caninum* nas amostras de solo positivas, indicando que os cães são a principal fonte de contaminação ambiental. Esses resultados fornecem dados de base sobre a contaminação do solo por ancilostomídeos na cidade de Hue e reforçam a necessidade de melhorar o manejo das fezes de cães, o saneamento e a educação em saúde pública.

## Introduction

Soil contamination with infective stages of zoonotic parasites in public areas represents an important route of human exposure, particularly in urban environments where frequent contact with contaminated ground occurs. Among soil-borne parasites, *Ancylostoma* spp. (hookworms) is of particular public health concern because their eggs and larvae can persist in soil and cause cutaneous larva migrans in humans following skin contact or accidental ingestion (Bethony et al., 2006). Public parks and playgrounds constitute high-risk environments for soil contamination, as they are commonly accessed by companion animals and intensively used by children, who are especially vulnerable to soil-transmitted infections. Dogs and cats infected with hookworms shed large numbers of eggs in their faeces, which contaminate the soil and subsequently develop into infective stages under favourable environmental conditions. Warm and humid climates typical of tropical and subtropical regions further enhance the survival and transmission of *Ancylostoma* spp. eggs and larvae in the environment (Lee et al., 2021; Nath et al., 2023).

Numerous studies conducted worldwide have reported considerable levels of soil contamination with *Ancylostoma* spp. eggs in public recreational areas. Tudor (2015) detected intestinal helminth eggs in 22% of soil samples collected from public parks in Bucharest. In Brazil, Cirne et al. (2017) reported *Ancylostoma* spp. egg contamination in 66.6% of public squares, while Mello et al. (2022) documented a contamination rate of 66.7% in playgrounds of primary schools. These findings indicate that environmental contamination with zoonotic hookworms is widespread in urban settings and may pose a substantial public health risk.

In Vietnam, canine hookworm infection has been reported at high prevalence levels, ranging from 52% to 96% in different regions (Le, 2005). *Ancylostoma* spp., particularly *A. caninum*, *A. braziliense*, and *A. ceylanicum*, are the main causative agents of cutaneous larva migrans (Bowman et al., 2010). The increasing ownership of dogs and cats, coupled with free-roaming practices and inadequate faeces management in public spaces, further elevates the risk of soil contamination and human exposure (Tylkowska et al., 2024; Rivera et al., 2025). Hue, the former imperial capital, is a well-known tourist city in Vietnam. Hue City is located in central Vietnam and characterized by a tropical monsoon climate with high humidity and distinct wet and dry seasons. The average annual temperature ranges from approximately 24 to 25°C, with peak temperatures exceeding 35°C during the summer months. The annual rainfall is relatively high, averaging 2,500–3,000 mm, with the majority occurring between September and December. Relative humidity is typically high, ranging from 80% to 90% throughout the year. The population density in Hue is estimated to be over 2,000 inhabitants per km^2^. Hue City is a major urban center where companion animal ownership has increased in recent years. Despite this, data on environmental contamination with *Ancylostoma* spp. eggs in public areas remain scarce. To date, no study has specifically assessed the prevalence of soil contamination with *Ancylostoma* spp. eggs in public parks and playgrounds in Hue City. Therefore, the present study aimed to determine the prevalence of soil contamination with *Ancylostoma* spp. eggs in parks and playgrounds in Hue City, Vietnam. The findings obtained from this research will provide an evidence-based foundation for government agencies to develop policies aimed at reducing environmental contamination with *Ancylostoma* spp. eggs.

## Material and Methods

### Soil sampling and processing

The required sample size was calculated using the two-stage cluster sampling (binary outcome) approach provided by resources from the website of Melbourne Veterinary School (Melbourne Veterinary School, 2025). The primary sampling units (PSUs) are parks/playgrounds; the secondary sampling units (SSUs) are soil samples. The parameters used for sample size estimation included an expected effect size of 0.5, a type I error rate (α) of 0.05 corresponding to a 95% confidence level, a type II error rate (β) of 0.2, and a desired study power (1 − β) of 0.8. According to local statistics, there are approximately 100 public parks and playgrounds in Hue City. For each parks/playgrounds, two pooled soil samples were collected, one from areas near trash bins and another from locations with high levels of human activity. Therefore, the PSUs and SSUs in this study are 100 and 2, respectively. Based on the sample size calculation, a minimum of 56 parks/playgrounds and 112 pooled samples were required. In this study, a total of 60 parks/playgrounds were included, and 120 pooled soil samples were collected per season.

A total of 240 soil samples were collected from public parks and playgrounds in Hue City during the two main climatic seasons: the dry season (March–August) and the rainy season (September–February of the following year), based on local climatic characteristics and average annual rainfall patterns. At each selected park or playground, soil samples were collected from two fixed locations: (i) areas surrounding public rubbish bins, where dog and cat faeces and household waste are likely to accumulate, and (ii) the main play areas or lawns, where residents and children frequently have direct contact with the soil. At each fixed location, soil was collected from five representative points and thoroughly homogenized to form a single composite sample. Specifically, for areas near waste bins, five subsampling points were evenly distributed around the bin. For playground areas, soil samples were collected from five points, including four at the corners and one at the center of the activity area.

To assess spatial differences in soil contamination with *Ancylostoma* spp. eggs across Hue City, the study area was stratified into two zones based on the degree of urbanization: an urban (central) zone and a peri-urban zone. The urban zone comprised 14 inner-city wards, characterized by high population density (>2,000 inhabitants/km^2^), dense housing, and a high concentration of public parks, playgrounds, and tourism- and commerce-related activities. In contrast, the peri-urban zone included wards located in the western and southern parts of the city, characterized by lower population density, more open spaces, and mixed land use, including vacant land, agricultural areas, and newly developed residential zones. Peri-urban (suburban) areas characterized by lower population density and mixed land use. This classification was based on differences in population density, land-use patterns, and urban infrastructure rather than strictly following administrative boundaries.

### Detection of *Ancylostoma* spp. eggs in soil samples

At each sampling point, surface vegetation was carefully removed, and topsoil was collected using a clean shovel from a depth of approximately 1–5 cm, with a minimum of 200 g collected per sample. The soil was placed into clearly labelled plastic bags indicating the sampling date and location, stored in insulated containers with ice packs, and transported to the Laboratory of the Faculty of Animal Science and Veterinary Medicine, Hue University of Agriculture and Forestry, for parasitological analysis.

Soil samples were processed using a modified sieving and centrifugal flotation method as described by Mizgajska-Wiktor (2005) and Santarém et al. (2009). A saturated sodium chloride (NaCl) solution with a specific gravity of approximately 1.18 was used for flotation. Briefly, 200 g of soil from each sample were soaked in a 1% Tween 80 solution for 30 minutes to facilitate egg detachment. The resulting suspension was sequentially filtered through sieves with mesh sizes of 1000 µm, 100 µm, and 45 µm. Material retained on the 45 µm sieve was washed into 50 mL Falcon tubes and centrifuged at 2000 rpm for 10 minutes, after which the supernatant was discarded. The sediment was resuspended in saturated sodium chloride (NaCl) solution to a final volume of 50 mL, thoroughly mixed, and centrifuged again under the same conditions. The supernatant was then transferred to a new tube, adjusted to 50 mL with saturated NaCl solution, mixed, and centrifuged once more at 2000 rpm for 10 minutes.

Following the final centrifugation, saturated NaCl solution was added to the tube until a convex meniscus formed, and a glass slide was placed in contact with the surface of the solution and left undisturbed for 15 minutes to allow egg flotation. The slide was then examined under a light microscope at 10× and 40× magnifications. Nematode eggs morphologically consistent with *Ancylostoma* spp. were identified based on the criteria described by Soulsby (1977). The level of soil contamination was semi-quantitatively assessed based on the number of *Ancylostoma* spp. eggs recovered per 200 g of soil using a modified Mcmaster chamber to improve analytical sensitivity (Daş et al., 2011). Following sieving and centrifugation procedures described above, the recovered sediment was resuspended and concentrated in saturated sodium chloride solution to obtain a final flotation suspension volume of 9 mL prior to egg counting using the modified McMaster technique. Each examination consisted of reading both chambers of the slide, corresponding to a total examined volume of 0.3 mL per reading. To increase the detection sensitivity, the reading procedure was repeated 10 times, resulting in a cumulative examined volume of 3 mL per sample. The number of *Ancylostoma* spp. eggs detected in all readings was recorded as n. The estimated number of eggs per 200 g of soil (EP200S) was calculated using the following formula: $EP200S=n\times \frac{9}{3}$(1)

The level of soil contamination was semi-quantitatively assessed based on the number of *Ancylostoma* spp. eggs recovered per 200 g of soil using a McMaster chamber (Daş et al., 2011. Contamination intensity was classified into three categories: low (+), corresponding to 1–4 eggs; moderate (++), corresponding to 5–10 eggs; and high (+++), corresponding to more than 10 eggs per 200 g of soil. This classification was used to facilitate comparison of contamination levels among sampling sites.

### Molecular identification of Ancylostoma caninum by PCR

After flotation, hookworm eggs from positive soil samples were recovered from the supernatant, centrifuged at 6,000 rpm for 5 minutes, and examined microscopically to confirm morphological compatibility with *Ancylostoma* spp. The resulting pellet was transferred to 1.5-mL Eppendorf tubes for molecular analysis.

To disrupt the eggshells and release genomic DNA, a heat-shock protocol adapted from De Oliveira et al. (2021) was applied. Briefly, 200 µL of the egg suspension were vortexed and centrifuged at 6,000 rpm for 5 minutes, after which the supernatant was discarded and replaced with nuclease-free water to a final volume of 200 µL. Samples were then subjected to alternating freeze–thaw cycles consisting of rapid freezing in liquid nitrogen followed by heating at 95 °C for 5 minutes. This procedure was repeated 10 times, with vortexing after each cycle to enhance eggshell disruption. The efficiency of egg rupture was randomly verified under light microscopy.

Genomic DNA was extracted using the phenol–chloroform method. The lysate was prepared by adding 400 µL of extraction buffer (50 mM Tris-HCl, pH 8.0; 50 mM EDTA; 1% SDS; 100 mM NaCl; 1% β-mercaptoethanol) and 10 µL of proteinase K, followed by incubation at 65 °C for 3 hours. After cooling to room temperature, 400 µL of phenol: chloroform: isoamyl alcohol (25:24:1) and 20 µL of 5 M NaCl were added, mixed thoroughly, and centrifuged at 13,000 rpm for 15 minutes. The aqueous phase was transferred to a new tube and further purified with chloroform: isoamyl alcohol (24:1) under the same centrifugation conditions.

DNA was precipitated by adding 20 µL of 3 M sodium acetate and two volumes of ice-cold absolute ethanol, followed by incubation at −20 °C for 30 minutes and centrifugation at 13,000 rpm for 15 minutes. The resulting pellet was washed with 500 µL of 70% ethanol, air-dried at room temperature for 30 minutes, resuspended in 50 µL of nuclease-free water, and stored at −20 °C until further use.

Extracted DNA was used as a template for PCR amplification targeting *Ancylostoma caninum*. PCR reactions were performed using FIREPol® Master Mix Ready to Load with 7.5 mM MgCl_2_, 5X (Solis BioDyne OU, Tartu, Estonia) following the manufacturer’s instructions, with the species-specific primers Ac-01-F (5′-TCGGGGAAGGTTGGGAGTAT-3′) and Ac-01-R (5′AGCAGTAAGGCGGCATTCAT-3′) described by Rehman et al. (2017). Thermal cycling conditions included an initial denaturation at 95 °C for 5 minutes; 35 cycles of denaturation at 95 °C for 25 seconds, annealing at 54 °C for 45 seconds, and extension at 72 °C for 2 minutes; followed by a final extension at 72 °C for 7 minutes 30 seconds. The positive control consisted of DNA extracted from hookworm eggs obtained from dog faeces, amplified using species-specific primers for *Ancylostoma caninum*, and the PCR product was confirmed by sequencing. The negative control consisted of nuclease-free water used in place of template DNA. PCR products were resolved by electrophoresis on 1.5% agarose gels stained with RedGel and visualised using a GelDoc imaging system.

### Data analysis

Data were entered and managed in Microsoft Excel 2016. Differences in the proportion of *Ancylostoma* spp.–positive sites across categories of explanatory variables were assessed using Fisher’s exact test in SPSS (SPSS Inc., Chicago, Illinois, USA), with *P* < 0.05 considered statistically significant. For each dichotomous variable, odds ratios (ORs) and 95% confidence intervals (95% CIs) were calculated using the reference category as the baseline (OR = 1.00). Site-level prevalence was calculated as the number of positive sites divided by the total number of sites surveyed. QGIS software was used to generate maps of sampling locations and to visualize the spatial distribution of *Ancylostoma* spp. egg contamination in the study area.

## Results

**Figure 1:**
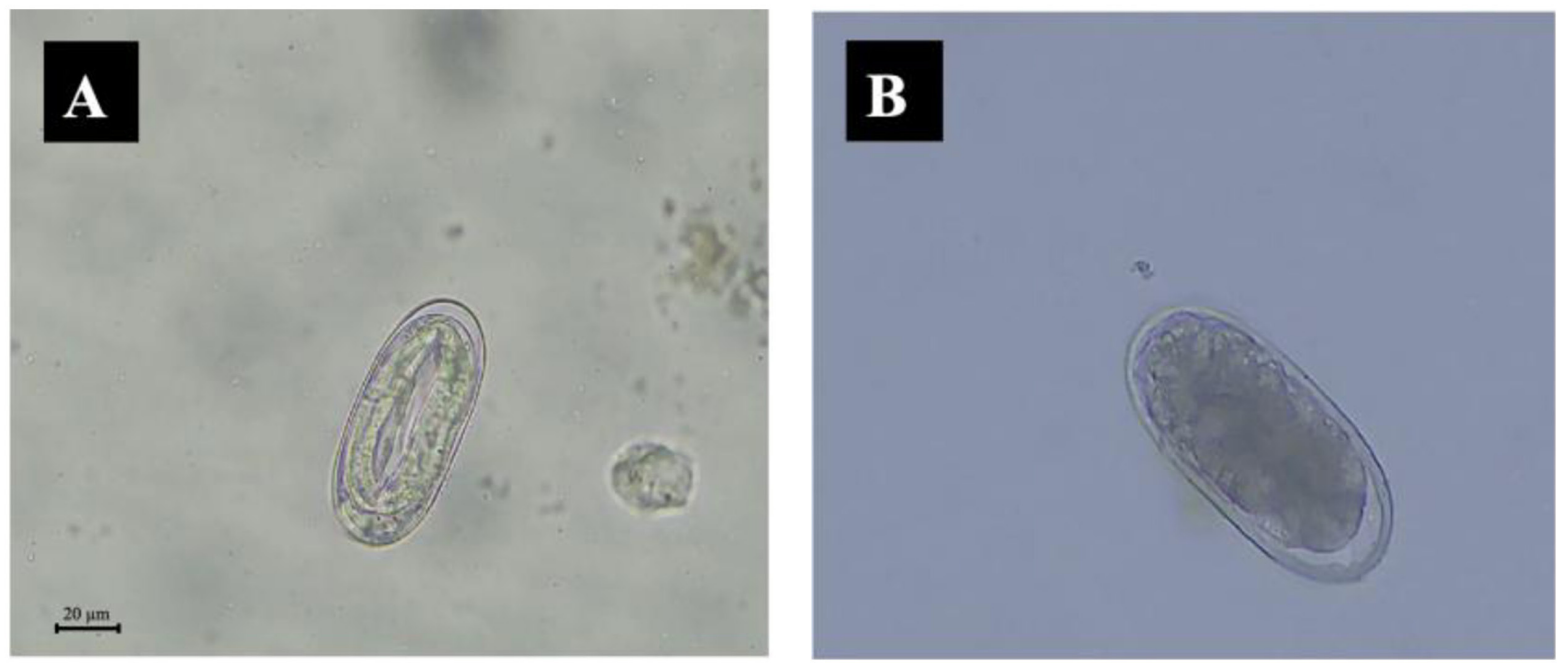
*Ancylostoma* spp. eggs in the soil examples. **A.** embryonated egg, 40x; **B.** unembryonated egg, 40x.

A total of 240 pooled soil samples (across two seasons) were collected from 60 public parks and playgrounds in Hue City. A park/playground was considered positive if at least one pooled sample tested positive for *Ancylostoma* spp. eggs, regardless of the sampling season or sampling location within the site. Based on this criterion, *Ancylostoma* spp. eggs were detected in 48 out of 60 parks/playgrounds, yielding an overall contamination rate of 80.0%. A representative microscopic image of a hookworm egg is shown in Figure 1

With respect to season, the proportion of positive sites was higher in the rainy season than in the dry season, at 71.67% (43/60) and 46.67% (28/60), respectively. Consistently, the odds of positivity in the dry season were lower than in the rainy season (OR = 0.35, 95% CI: 0.16–0.74). When comparing sampling positions within parks and playgrounds, the proportion of positive sites did not differ significantly between playground areas and areas surrounding trash bins (OR = 1.24, 95% CI: 0.59 – 2.62). The proportion of positive sites was higher in the central than in the peri-central zone (93.10% vs. 67.74%), with higher odds of positivity in the central zone (OR= 6.41, 95% CI: 1.27 – 32.26) (Table 1).

**Table 1:** Prevalence of *Ancylostoma* spp. eggs in soil from public parks and playgrounds in Hue City, Vietnam.

**Categories**		**No. of samples**	**Positive samples**	**Positive rate (%)**	**OR**	**95% CI**
Season	Dry season	60	28	46.67	0.35	0.16-0.74
	Rainy season	60	43	71.67	1.00	
Urban zone	Central	29	27	93.10	6.41	1.27-32.26
	Peri-central	31	21	67.74	1.00	
Sampling location	Playground	60	40	66.67	1.24	0.59-2.62
	Garbage bin area	60	37	61.67	1.00	

Note: No. = number

**Figure 2:**
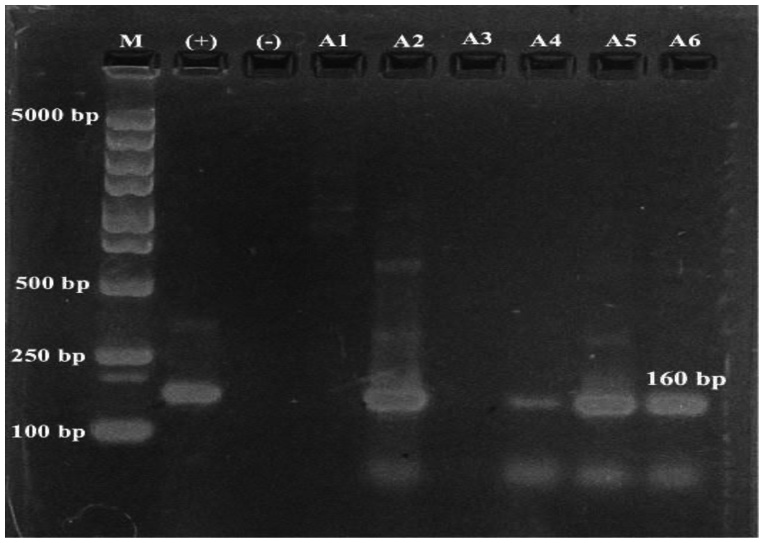
PCR amplification products of *Ancylostoma caninum* in agarose gel. Lane M: Ladder; lane (+): positive control; lane (−): negative control; lanes A1–A6: samples.

All soil samples that tested positive for hookworm eggs by flotation were subjected to conventional PCR targeting *Ancylostoma caninum*. Amplification using species-specific primers produced the expected band, confirming the presence of *A. caninum* DNA in the examined soil samples (Figure 2).

Regarding contamination intensity, 48 of the 60 parks and playgrounds surveyed had at least one positive soil sample. Most of these positive sites showed low egg counts: 39/48 (81.25%) were classified as having low contamination (1–4 eggs/200 g of soil), 8/48 (16.67%) as moderate contamination (5–10 eggs/200 g of soil), and only 1/48 (2.08%) as high contamination (>10 eggs/200 g of soil) (Table 2).

**Table 2:** Intensity of *Ancylostoma* spp. eggs contamination in soil samples collected from public parks and playgrounds in Hue City (n=48).

**Contamination level**	**No. of eggs (eggs/200g soil)**	**No. of positive sites**	**Positive rate (%)**	**Mean ± SD**
**+**	**1- 4**	**39**	**81.25**	**2.28 ± 1.19**
**+ +**	**5-10**	**8**	**16.67**	**6.0 ± 1.41**
**+ + +**	**>10**	**1**	**2.08**	

Note: No. = number

**Figure 3:**
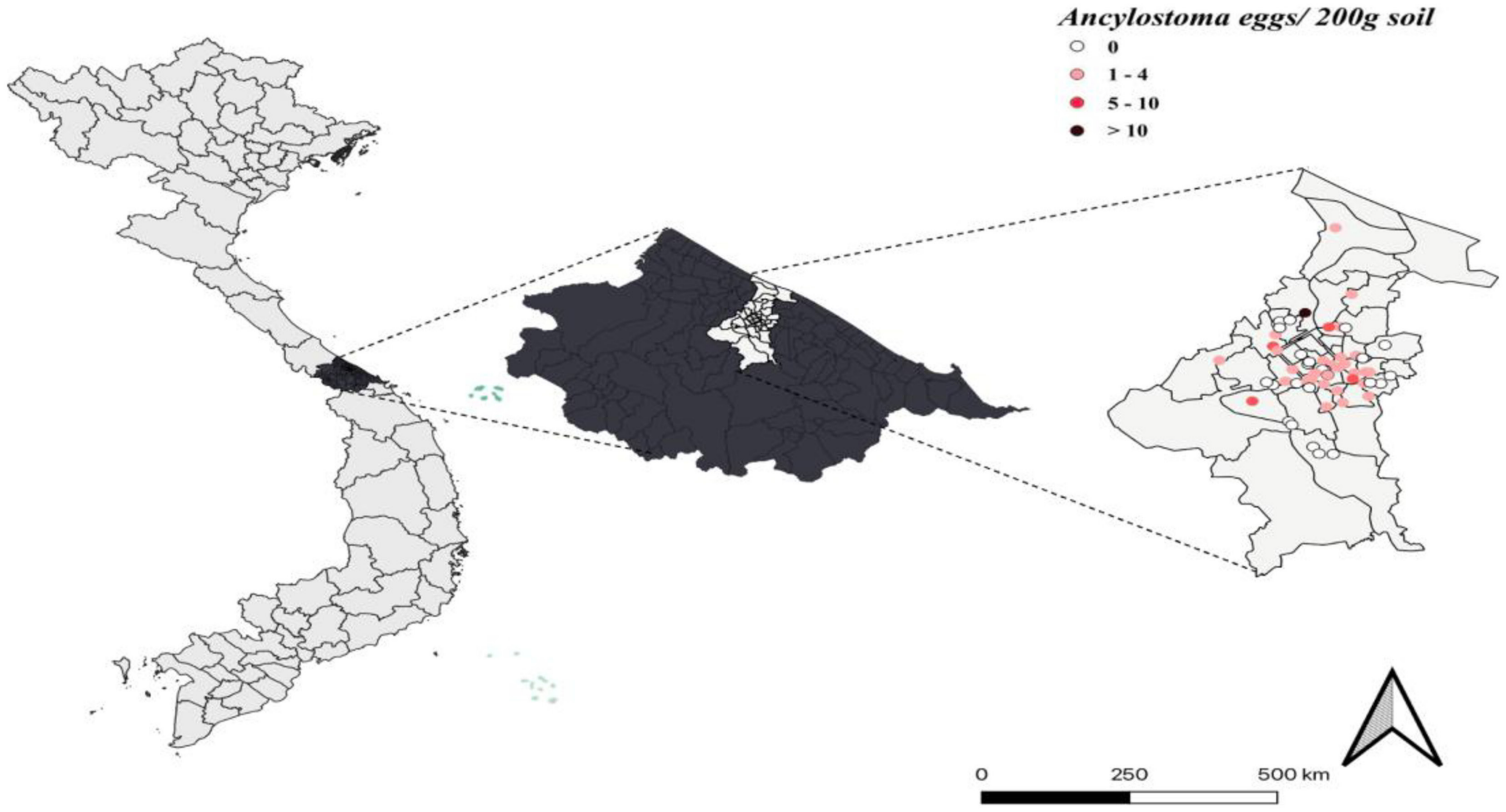
Map of the distribution and intensity of *Ancylostoma* spp. eggs contamination at 60 parks and playgrounds in Hue City. Each dot represents one sampling site; colours indicate mean contamination intensity (eggs/200 g of soil): 0 (negative), 1–4 (low), 5–10 (moderate), and >10 (high).

The map of contamination prevalence and intensity across the 60 parks and playgrounds showed that positive sites were widely distributed throughout Hue City but tended to cluster more densely in the central urban area. In contrast, negative sites or those with only low-level contamination were recorded more frequently in the peri-central zone (Figure 3).

## Discussion

This study represents the first survey to assess soil contamination in public parks and playgrounds in Hue City with zoonotic parasites, particularly *Ancylostoma* spp., likely originating from dog and cat faeces. This finding is consistent with previous parasitological studies on dogs in Vietnam, in which *A. caninum* has been reported as a common species with substantial prevalence in several regions, including central Vietnam (Ng-Nguyen et al., 2015). Although this species does not complete its life cycle in humans, its larvae can penetrate the skin, causing dermatitis, pruritus, and cutaneous larva migrans (Prociv & Croese, 1996). Children and individuals who frequently have direct contact with soil in parks and playgrounds, therefore, represent important risk groups (Mohd Zain et al., 2015). Among the 60 parks and playgrounds surveyed, 48 sites were positive for *Ancylostoma* spp. eggs (80.0%), indicating a noteworthy level of soil contamination in recreational public areas in Hue. This proportion is comparable to that reported from other tropical environments, such as Malaysia (88.3%) (Mohd Zain et al., 2015), but substantially higher than that observed in temperate countries such as Poland, where contamination rates were 5.0% in Wrocław and 0% in Lublin (Kuśmierek et al., 2020; Bojar & Kłapeć, 2012). These geographic differences underscore the predominant influence of climate on the survival of hookworm eggs and larvae in the environment. *Ancylostoma* spp. is mainly endemic in tropical and subtropical regions, where high temperatures and humidity promote egg development and long-term persistence of infective L3 larvae (Mabaso et al., 2003; Hotez et al., 2004; Loukas et al., 2016). Hue City, with its humid tropical monsoon climate, high rainfall, and persistent humidity, provides favourable ecological conditions for the maintenance of infective stages in soil on a broad scale. In addition to climatic factors, dogs and cats infected with hookworms are the primary source of faecal contamination in the environment, particularly in the absence of systematic faeces removal and effective pet control in public areas (Tylkowska et al., 2024).

The degree of urbanization also appeared to influence contamination levels. The higher prevalence of hookworm eggs in central areas may partly reflect differences in dog and cat density and in the intensity of park use compared with peri-central zones. Central Hue has a high human population density and a greater number of pet-owning households, likely resulting in larger amounts of dog and cat faeces being deposited in public spaces, where faeces are not always removed. In Naples (Italy), Rinaldi et al. (2006) demonstrated a positive correlation between the amount of dog faeces and human population density (p < 0.001), with the most densely populated areas showing the highest levels of canine faecal contamination; 2.4% of dog faecal samples contained *Ancylostoma caninum* eggs. Socioeconomic conditions have also been associated with the extent of faecal and parasitic contamination. In Buenos Aires (Argentina), Rubel & Wisnivesky (2005) reported that the density of dog faeces in low-income neighbourhoods was higher than in middle-income areas (p < 0.05). Moreover, the prevalence of hookworm eggs in dog faeces reached 53% in low-income neighbourhoods, compared with 14% in middle-income areas (p < 0.01).

Seasonal patterns were also evident in the present study, with a higher proportion of positive sites in the rainy season than in the dry season (71.67% vs. 46.67%; p = 0.009). This is consistent with the biology of *Ancylostoma* spp., as eggs and larvae require adequate moisture and suitable temperatures for development and survival. In the Philippines, Horiuchi et al. (2013) likewise observed higher rates of soil contamination with parasites during the rainy season. However, other studies have shown that seasonal patterns may vary according to local climate and rainfall characteristics. In Fortaleza (northeastern Brazil), Melo et al. (2020) reported a higher proportion of positive soil samples in the dry season (96.7%) than in the rainy season (68.3%), suggesting that heavy rainfall may wash eggs away from the soil surface. In contrast, in Macapá (northern Brazil), an equatorial region with high rainfall throughout the year, parasite loads in soil were higher in the rainy season. These differences indicate that the impact of seasonality depends not only on total rainfall but also on rainfall patterns, soil characteristics, sanitation conditions, and community behaviour. In the dry season, environmental cleaning and collection of pet faeces may be easier, with faeces drying and becoming less widely dispersed. Conversely, during the rainy season, cleaning becomes more difficult, and dog and cat faeces are more likely to be dispersed and infiltrate moist soil, favouring egg development and accumulation. The observed effects of season and urban zone in our study thus reflect the combined influence of environmental conditions and sources of contamination on the presence of hookworm eggs in soils of parks and playgrounds.

Despite the high proportion of positive sites, most contaminated locations exhibited only low contamination intensity (65%), suggesting widespread but generally low-density contamination. One possible explanation is that the prolonged rainy season in Hue facilitates the movement of contaminated soil across different areas within each park. Rainwater may redistribute soil containing hookworm eggs, thereby reducing the number of eggs in the specific 200 g sample collected at each point while expanding contamination across the park as a whole. This may account for the lack of significant differences in contamination between sampling positions and supports the view that contamination is diffuse rather than localized. In addition to the predominance of low-intensity contamination, the study also identified a smaller number of “hotspots” with moderate (13.33%) and high (1.67%) levels of contamination. These hotspots warrant more intensive intervention than other locations, allowing targeted control measures that are easier to monitor and more cost-effective than citywide interventions. At present, there is no international standard for categorizing light, moderate, and heavy contamination with *Ancylostoma* spp. eggs in soil, which complicates comparisons of intensity across studies. Furthermore, no standardized methods exist for sampling, recovery, and quantification of soil-transmitted helminth eggs in soil; many authors have acknowledged that heterogeneity in sampling and laboratory procedures is a major obstacle for research on STHs (Steinbaum et al., 2017). The use of different methodologies inevitably leads to substantial variability in reported egg counts. Consequently, the thresholds used in the present study are primarily intended for internal comparisons and for guiding local prioritization of interventions.

The absence of significant differences between sampling positions within parks may also be related to patterns of space use and the behaviour of dogs and cats. All sampling points within a given park are exposed to the same local climatic conditions, and thus similar environmental conditions for the survival and development of hookworm eggs and larvae would be expected. In addition, the extended rainy season in Hue not only provides favourable moisture for *Ancylostoma* egg and larval development but also contributes to the redistribution of contaminated soil throughout the park. None of the surveyed parks had designated dog toileting areas, so dogs and cats can defecate in multiple locations, resulting in contamination across all areas. This observation is consistent with the findings of Steinbaum et al. (2016) in rural Kenya, where differences in STH contamination between household sampling locations (e.g. house entrance, latrine entrance) were not statistically significant (p = 0.41), despite some variation in prevalence. In contrast, another study found that areas around rubbish bins had the highest proportion of soil samples positive for hookworm eggs (18.8%), hypothesizing that free-roaming dogs and cats congregate near bins in search of food scraps (Shad et al., 2024). In Hue, improper disposal of household waste persists in some parks. During fieldwork, we observed household garbage not only around bins but also scattered in various locations. These informal rubbish points may attract free-roaming dogs and cats, which can then disseminate *Ancylostoma* spp. eggs over a wider area beyond the vicinity of the bins, thereby reducing the ability to detect positional differences in contamination. Nonetheless, this explanation remains hypothetical and should be tested in future studies that quantitatively record waste distribution and dog and cat movements in parks or that compare contamination rates between parks with and without informal rubbish points. Differences in sampling methodology (e.g. soil mass, sampling depth) and in the classification of sampling positions between studies may further contribute to the apparent inconsistency. These factors should be taken into account when comparing the present results with those from other urban contexts.

From a public health and urban veterinary management perspective, the findings indicate that soils in parks and playgrounds in Hue may constitute a potential source of exposure for the community, particularly for children and individuals who frequently have direct contact with the ground. Although contamination intensity at most sites was low, the widespread presence of *Ancylostoma* spp. eggs, together with a subset of sites with higher contamination, suggests that the risk of transmission in the urban environment is ongoing. Given that hookworm transmission is closely linked to both animal hosts and environmental conditions, local control strategies should adopt a One Health approach, combining improved management of pet faeces, control of free-roaming dogs and cats, and regular deworming of companion animals with environmental sanitation measures. Prioritizing regular surveillance in parks with higher contamination levels, particularly during the rainy season, could help optimize resource allocation and support the evaluation of intervention effectiveness over time.

This study has some limitations that should be noted. The analysis focused on the detection of *Ancylostoma* spp. eggs in soil samples and did not include the recovery of larval stages. Under favourable environmental conditions, hookworm eggs can develop rapidly into infective L3 larvae within 5–10 days. Therefore, the number of eggs detected in soil samples may be lower than the actual level of contamination, which could have influenced the results.

## Conclusions

The present study showed that soil in public parks and playgrounds in Hue city is highly contaminated with *Ancylostoma* spp. eggs (80%), particularly in central urban areas and during the rainy season. Conventional PCR confirmed the presence of *Ancylostoma caninum* in positive soil samples, supporting the role of dogs as the main source of environmental contamination and highlighting the epidemiological risk to public health, especially for children. Targeted measures are needed to improve the management of dog and cat faeces, enhance environmental sanitation, and raise pet owners' awareness of the risk of parasite transmission from contaminated environments.

## Data Availability

The datasets generated during the current study are not publicly available due to ethical and regulatory restrictions but are available from the corresponding author upon reasonable request.
